# Incidence, Clinical Features and Impact on Anti-Tuberculosis Treatment of Anti-Tuberculosis Drug Induced Liver Injury (ATLI) in China

**DOI:** 10.1371/journal.pone.0021836

**Published:** 2011-07-05

**Authors:** Penghui Shang, Yinyin Xia, Feiying Liu, Xiaomeng Wang, Yanli Yuan, Daiyu Hu, Dehua Tu, Yixin Chen, Peiyuan Deng, Shiming Cheng, Lin Zhou, Yu Ma, Lizhen Zhu, Weiwei Gao, Hongyuan Wang, Dafang Chen, Li Yang, Pingping He, Shanshan Wu, Shaowen Tang, Xiaozhen Lv, Zheng Shu, Yuan Zhang, Zhirong Yang, Yan Chen, Na Li, Feng Sun, Xiaoting Li, Yingjian He, Paul Garner, Siyan Zhan

**Affiliations:** 1 Department of Epidemiology and Bio-Statistics, School of Public Health, Peking University Health Science Centre, Beijing, China; 2 Center for Tuberculosis Control and Prevention, Chinese Center for Disease Control and Prevention, Beijing, China; 3 Center for Disease Control and Prevention in Guangxi Zhuang Autonomous Region, Nanning, China; 4 Center for Disease Control and Prevention in Zhejiang Province, Hangzhou, China; 5 Center for Disease Control and Prevention in Jilin Province, Changchun, China; 6 Center for Disease Control and Prevention in Chongqing Municipality, Chongqing, China; 7 Beijing Institute for Tuberculosis Control, Beijing, China; 8 Center for Drug Reassessment, State Food and Drug Administration, Beijing, China; 9 Beijing Tuberculosis and Thoracic Tumor Research Institute, Beijing, China; 10 Department of Public Health, School of Medicine, Indiana University, Indianapolis, United States of America; 11 Center for Disease Control and Prevention in Fangshan District, Beijing, China; 12 Beijing Institute for Cancer Research, Beijing, China; 13 International Health Group, Liverpool School of Tropical Medicine, Liverpool, United Kingdom; San Francisco General Hospital, University of California San Francisco, United States of America

## Abstract

**Background:**

Anti-tuberculosis drug induced liver injury (ATLI) is emerging as a significant threat to tuberculosis control in China, though limited data is available about the burden of ATLI at population level. This study aimed to estimate the incidence of ATLI, to better understand its clinical features, and to evaluate its impact on anti-tuberculosis (TB) treatment in China.

**Methodology/Principal Findings:**

In a population-based prospective study, we monitored 4,304 TB patients receiving directly observed treatment strategy (DOTS) treatment, and found that 106 patients developed ATLI with a cumulative incidence of 2.55% (95% Confidence Interval [CI], 2.04%–3.06%). Nausea, vomiting and anorexia were the top three most frequently observed symptoms. There were 35 (33.02%) ATLI patients with no symptoms, including 8 with severe hepatotoxicity. Regarding the prognosis of ATLI, 84 cases (79.25%) recovered, 18 (16.98%) improved, 2 (1.89%) failed to respond to the treatment with continued elevation of serum alanine aminotransferase, and 2 (1.89%) died as result of ATLI. Of all the ATLI cases, 74 (69.81%) cases changed their anti-TB treatment, including 4 (3.77%) cases with medication administration change, 21 (19.81%) cases with drugs replacement, 54 (50.94%) cases with therapy interruption, and 12 (11.32%) cases who discontinued therapy. In terms of treatment outcomes, 53 (51.46%) cases had TB cured in time, 48 (46.60%) cases had therapy prolonged, and 2 (1.94%) cases died. Compared with non-ATLI patients, ATLI patients had a 9.25-fold (95%CI, 5.69–15.05) risk of unsuccessful anti-TB treatment outcomes and a 2.11-fold (95%CI,1.23–3.60) risk of prolonged intensive treatment phase.

**Conclusions/Significance:**

ATLI could considerably impact the outcomes of anti-TB treatment. Given the incidence of ATLI and the size of TB population in China, the negative impact is substantial. Therefore, more research and efforts are warranted in order to enhance the diagnosis and the prevention of ATLI.

## Introduction

Tuberculosis (TB) continues to be a major health problem, with 9.4 million incident cases and 1.7 million deaths globally in 2009 [1]. China ranked second on the list of the top five TB endemic countries in terms of incident cases: India, China, South Africa, Nigeria and Indonesia. China (1.1–1.5 million) and India (1.6–2.4 million) combined accounted for 35% of all TB cases worldwide in 2009 [1]. In view of the seriousness of the problem, China established the China National Tuberculosis Prevention and Control Scheme (CNTS) in 1990 and has been implementing directly observed treatment strategy (DOTS) since 1991, which constitutes the cornerstone of the current strategy for TB control and covers the entire population of China [2], [3]. The key component of DOTS strategy is the standard anti-TB short course chemotherapy regimen, which requires continually taking drug combinations of Isoniazid (H), Rifampicin (R), Pyrazinamide (Z), Ethambutol (E) and Streptomycin (S) every other day for 6–9 months [4].

Although these anti-TB drugs have shown that they are able to contain and kill *Mycobacterium tuberculosis* effectively, they are known to induce various adverse effects, including liver injury, skin reactions, gastrointestinal and neurological disorders [5], [6]. Anti-tuberculosis drug induced liver injury (ATLI) is one of the most important and serious adverse effects [7], [8], which accounts for more than 7.0% of the overall adverse effects [9], [10]. The incidence of ATLI during standard multi-drug TB treatment has been reported varying from 2.0% to 28.0% according to different populations and definitions [7]. Moreover, ATLI diminishes the effectiveness of anti-TB treatment, as they may cause non-adherence, and further leads to treatment failure, recurrence or the emergence of drug-resistance [7], [11], [12]. These negative consequences could significantly impair the overall effects of TB epidemic control.

In order to identify the adverse events earlier and provide timely intervention, it is critical to understand the clinical features of ATLI, such as the time of onset, severity, common clinical symptoms, and its potential outcomes. Despite DOTS two decades of implementation in China the CNTS does not include detailed standard operating procedures regarding diagnosis and management of possible ATLI. Therefore, we conducted this population-based prospective study among patients with positive TB smear and those who have received DOTS treatment. The aim of this study was to estimate the incidence of ATLI, better understand its clinical features, and more importantly, to evaluate its impact on anti-TB treatment. We believe that the findings of this study will provide additional clinical guidance to improve TB treatment and enhance monitoring and control of the TB epidemic in China.

## Methods

The prospective study was approved by the Ethics Committee of Center for Tuberculosis Control and Prevention of China. Written informed consent was obtained from every participant or surrogate before enrolment.

### Patient's enrollment

This study consists of a cohort of patients with pulmonary TB and who have received DOTS treatment in four geographically and economically diverse areas of China. This cohort was previously established in the study entitled ‘Anti-tuberculosis Drugs induced Adverse Reactions in China National Tuberculosis Prevention and Control Scheme Study’ (ADACS) [2]. In brief, between October 2007 and June 2008, 6,460 smear-positive patients who receive standard short-course chemotherapy recommended by CNTS were initially identified in 52 counties of 4 provinces using the stratified, cluster and probability proportional to size (PPS) sampling method. Among them, 155 patients did not meet the study inclusion criteria and 1,817 patients did not respond to the study. Therefore, a total of 4,488 patients were enrolled into the cohort. The differences in terms of age and sex between the 4,488 remainders and 1,817 non-responders were not statistically significant (Data not shown).

### Investigation and monitoring

At the enrollment, eligible patients were interviewed with a questionnaire that collects information on demography, medical history, co-morbidities, diagnosis of TB, previous anti-TB treatment and current use of medication. During the study, the patients were asked to take a list of laboratory examinations prior to the initiation of anti-TB treatment, and repeat again during 1 to 2 months following the initiation of treatment. The lab examinations included blood and urine routine tests, serum alanine aminotransferase (ALT), aspartate aminotransferase (AST) and total bilirubin (TBil) tests, renal function tests, and hepatitis B surface antigen (HBsAg) tests. For those patients with suspected ATLI, a third examination was conducted. All tests were completed at the local decentralized laboratories of Center for Disease Control and Prevention (CDC) in China using uniform reagents and equipments corrected with standard sample from China national CDC laboratory.

Patients receiving primary TB treatment were monitored for 6 months, while patients with re-treatment TB were monitored for 8 months. The first 2-month period was defined as the intensive treatment phase, in which primary and re-treatment patients took HRZE and HRZES combination, respectively. The subsequent 4 or 6-month period was defined as the consolidation treatment phase, in which patients took HR and HRE combination respectively. According to CNTS, patients whose status does not change to negative for smear results during the intensive treatment phase needed to extend the intensive phase by 1 month.

During the monitoring period, patients were instructed to fill in the ADACS calendars, which were designed to record patients' self-reported sign/symptoms and drug usages. The listed feelings were discomforts, including nausea, vertigo, headache, diarrhea, arthralgia, paresthesia, visual and auditory abnormal feelings. Meanwhile, the supervising doctors monitored patients for hepatotoxicity symptoms according to DOTS and check the ADACS calendars every other day. The patients were asked to report to their supervising doctors once they could no longer bear the discomfort as a result of treatment or had symptoms of hepatitis (such as nausea, anorexia, vomiting, diarrhea, icterus, etc.). If the supervising doctors suspected the cause was ATLI, the patients would be referred to CDC doctors for further examinations and ATLI investigations. The ATLI investigation procedure would be initiated as well if patients had abnormal lab results within 1 to 2 months following the anti-TB treatment, even if patients did not experience any notable symptoms.

The Center for Drug Reassessment (CDR) of Chinese State Food and Drug Administration was responsible for adjudicating all the suspected ATLI cases, which were submitted online by each study site. National Adverse Drug Reactions Monitoring system of the CDR checked the report, evaluated the causality of drugs and events, and confirmed the ATLI status of the patient. During the adjudication process, the causality assessment followed the standards developed by WHO Uppsala Monitoring Centre system [2] which classifies the association as certain, probable/likely, possible, unlikely, conditional/unclassified or inaccessible/unclassifiable.

Once a suspected ATLI was identified, the investigator would trace the patient's ATLI treatment, prognosis, and clinical features, as well as the outcome of anti-TB treatment. In this study, Anti-TB treatment outcomes were recorded and classified into two categories: successful outcomes (defined as the completion of treatment and patients being cured) and unsuccessful outcomes, including treatment failure, deaths of patients, default or patients being transferred out [13].

### Assessment of ATLI

In this study, the criteria for ATLI diagnosis was not only based on the definition of hepatotoxicity developed by American Thoracic Society (ATS) [14], but also took into account the various definitions published in previous studies [7], [15]–[17], as well as the diagnosis criteria developed by the CDR of Chinese State Food and Drug Administration. Specifically, ATLI was diagnosed when there is an increase in serum ALT or AST that was greater than three times of the upper limit of normal (ULN), or in TBil greater than two times of the ULN, when other causes were excluded: new viral hepatitis infections, other potentially hepatotoxic medications that would confound the picture, alcohol consumption, other liver diseases, and/or drug use. All assessments were double checked by two experienced physicians. The severity of hepatotoxicity was classified according to the WHO Toxicity Classification Standards [7]. Based on this definition, severity of hepatotoxiciy was considered as follow: mild (3 times ULN<ALT or AST≤5 times ULN, or 2 times ULN<TBil≤5 times ULN) and severe (ALT or AST or TBil>5 times ULN).

### Statistical methods

The baseline characteristics of participants were described as median (inter-quartile range, IQR) for continuous variables (not subject to normal distribution), and percentages for categorical variables.

The cumulative incidence was calculated using Kaplan Meier method to deal with the censored data. Clinical features and impact on anti-TB treatment of ATLI were reported using descriptive analysis. In this study, the estimated incidence of ATLI was standardized by age and sex using two reference populations [2]: one population from the 4th national TB epidemiology investigation of China in 2000, and the other from national TB epidemic surveillance database of 2008.

The potential impact of ATLI on the length of intensive treatment period, the anti-TB treatment effects and clinical outcome were assessed with relative risk (RR), attributable risk proportion (AR%) and population attributable risk proportion (PAR%). AR% is the percent of the incidence of an outcome in the exposed that is due to the exposure. PAR% is the percent of the incidence of an outcome in the population including both exposed and non-exposed population that is due to exposure.

Because this study was designed to allocate the same number of patients in each province and then did PPS sampling in county level, a sample weight was used in the calculation and comparison of ATLI incidence rate. Sample weight was calculated in SPSS procedure of weight according to total TB patients in each province in 2006, which was also used in the PPS sampling.

The statistical analyses were performed using SPSS for Windows (version 13.0; SPSS Inc.). A two-sided P value less than 0.05 was set as the significant level.

## Results

### Characteristics of participants

In the 4,488 recruited patients, 129 patients dropped out during the monitoring, 23 patients died due to TB, and 32 died due to other reasons including heart disease, cancer and accidents. As a result, 4,304 patients completed the study, with a median observation time of 184 days. There were no statistically significant differences in terms of age and sex between the 4,304 participants and the 129 patients who dropped out (Data not shown). The characteristics of 4,304 patients at baseline were shown in Table 1. All 4,304 participants received at least 2 times of the ALT, AST and TBil tests. After starting anti-TB treatment, 206 patients had greater than 1 time of UNL elevation of ALT, 58 patients had greater than 1 time of UNL elevation of AST, and 9 patients had greater than 1 time of UNL elevation of TBil (Table 2).

**Table 1:** Baseline characteristics of 4304 patients with tuberculosis smear positive.^a^

Category	Subcategory	Result
Age, median years (IQR)		42.00 (29.00–55.00)
Male sex, number (%)		3082 (71.60%)
Weight, median kg (IQR)		52.70 (48.00–58.00)
Education status, number (%)	None/elementary school	1895 (44.03%)
	Secondary school	2260 (52.51%)
	College/higher	135 (3.14%)
	missing	14 (0.32%)
Type of treatment, number (%)	Primary treatment tuberculosis	3556 (82.62%)
	Re-treatment tuberculosis	748 (17.38%)
HBsAg, number (%)	Positive	469 (10.89%)
	Negative	3613 (83.95%)
	Unknown	222 (5.16%)
History of other diseases, number (%)	Diabetes	51 (1.18%)
	Liver disease^b^	17 (0.40%)
	Biliary tract disease^c^	6 (0.14%)
	Nephropathy	17 (0.40%)
	Gastroenteropathy	40 (0.93%)
	Others	103 (2.40%)

a Abbreviation used in table: IQR, inter quartile range.

b Including hepatitis B/C, alcohol liver disease, hepatapostema, hepatic cyst, hepatocirrhosis and schistosomiasis.

c Including cholecystitis and cholelithiasis.

**Table 2:** Elevation of ALT/AST and TBil after starting anti-TB treatment in TB patients by grade.^a^

Grade	Relative to ULN	No. of patients	Percentage(%)
ALT			
1	**>1 times the ULN**	58	28.15
2	**>2 times the ULN**	59	28.64
3	**>3 times the ULN**	49	23.79
4	**>5 times the ULN**	40	19.42
Sub-total		206	100.00
AST			
1	**>1 times the ULN**	32	55.17
2	**>2 times the ULN**	18	31.04
3	**>3 times the ULN**	7	12.07
4	**>5 times the ULN**	1	1.72
Sub-total		58	100.00
TBil			
1	**>1 times the ULN**	0	0.00
2	**>2 times the ULN**	6	66.67
3	**>3 times the ULN**	3	33.33
4	**>5 times the ULN**	0	0.00
Sub-total		9	100.00

a Abbreviation used in table: ALT, alanine aminotransferase; AST, aspartate aminotransferase; TBil, total bilirubin; ULN, upper limit of normal.

### Incidence and clinical features of ATLI

Among patients with abnormal ALT, AST or TBil level, 106 were diagnosed with ATLI, and the cumulative incidence of ATLI was 2.55% (95% Confidence Interval [CI], 2.04%–3.06%). The incidence of ATLI was 2.58% and 2.42% respectively after being standardized by age and sex using standard population.

Of the 106 ATLI cases, further causality assessment revealed that 17 cases (16.04%) were identified as certain, 65 (61.32%) as probable and 24 (22.64%) as possible. 71.59 percent of ATLI cases were identified within 2 months after starting anti-TB treatment. The median interval in days between the initiation of TB treatment and the detection of ATLI was 52.50 (IQR:30.00, 63.00) for all patients, 42.50 (IQR:29.25, 61.75) for primary and 61.00 (IQR:53.25, 68.25) for re-treatment TB patients. The difference between primary and re-treatment TB patients was not statistically significant (P = 0.29).

In terms of the severity of ATLI, 65 (61.32%) cases had mild hepatotoxicity and 41 (38.68%) had severe hepatotoxicity. There was no significant difference with respect to the severity of ATLI between primary and re-treatment TB patients (P = 0.30).

Among the 106 ATLI cases, nausea (41.51%), vomiting (39.62%) and anorexia (24.53%) were the top three most frequently reported symptoms (Table 3). 35 patients (33.02%), including 27 with mild hepatotoxicity and 8 patients with severe hepatotoxicity, did not have any notable clinical symptoms. There was no significant difference in terms of demographic characteristics between patients with and without clinical symptoms (P>0.05).

**Table 3:** The frequency of symptoms in 106 ATLI cases.^a^

Symptoms	Frequency (%)
Nausea	44 (41.51)
Vomiting	42 (39.62)
Anorexia	26 (24.53)
Dizziness	26 (24.53)
Abdominal symptoms (abdominal pain/discomfort/diarrhea)	24 (22.64)
Rash/pruritus	20 (18.87)
Fatigue	19 (17.92)
Icterus/jaundice	4 (3.77)
Dark urine	1 (0.94)
No clinical symptoms	35 (33.02)

a Abbreviation used in table: ATLI, Anti-tuberculosis Drug Induced Liver Injury.

In all the 106 ATLI cases, 88 (83.02%) sought medical advice. 78 (73.58%) received further medical examinations, 85 (80.19%) received medical treatment and 17 (16.04%) were hospitalized. Regarding the prognosis of ATLI, 84 cases (79.25%) recovered (ALT test turned normal, and clinical symptoms disappeared), 18 (16.98%) improved (ALT test turned normal, and clinical symptoms improved), 2 (1.89%) patients failed to the treatment with continued abnormal ALT, and 2 patients (1.89%) died of ATLI.

When comparing the 17 ATLI in-patients (including 2 died in hospital) with the 89 ATLI patients that were not hospitalized, it was found that the patients who were required for hospitalization had an earlier onset t (35 days vs. 56 days), although the difference was not significant (P = 0.21) (Table 4). After the start of anti-TB treatment, a higher percentage of in-patients presented symptoms, particularly anorexia, as compared to that in those without hospitalization (94.1% vs. 61.8%) (P = 0.01).

**Table 4:** The pattern of 17 ATLI in-patients^a^ and 89 non-hospitalized ATLI patients in 106 ATLI cases.^b^

Category	Subcategory	ATLI in-patients	non-hospitalized ATLI patients	P value
ATLI onset time, median days (IQR)		35.00 (25.50,58.50)	56.00 (30.00–63.50)	0.21
ALT	>5 times the ULN, number (%)	10 (62.5)	30 (41.1)	0.12
	≤5 times the ULN, number (%)	6 (37.5)	43 (58.9)	
AST	>5 times the ULN, number (%)	0 (0.0)	1 (12.5)	-
	≤5 times the ULN, number (%)	0 (0.0)	7 (87.5)	
TBil	>5 times the ULN, number (%)	0 (0.0)	0 (0.0)	-
	≤5 times the ULN, number (%)	1 (100.0)	8 (100.0)	
With symptom, number (%)		16 (94.1)	55 (61.8)	0.01
	Nausea, number (%)	11 (68.8)	28 (50.9)	0.21
	Vomiting, number (%)	10 (62.5)	27 (49.1)	0.35
	Anorexia, number (%)	10 (62.5)	19 (34.6)	0.04

a 17 ATLI in-patients included 2 died of ATLI.

b Abbreviation used in table: ATLI, Anti-tuberculosis Drug Induced Liver Injury; ALT, alanine aminotransferase; AST, aspartate aminotransferase; TBil, total bilirubin; ULN, upper limit of normal.

### Impact on anti-TB treatment of ATLI

Of all the 106 ATLI cases, 74 (69.81%) cases changed their anti-TB treatment, including 4 (3.77%) with changes in medication administration, 21 (19.81%) had drugs replacement, 54 (50.94%) interrupted the TB treatment, and 12 (11.32%) discontinued the TB treatment. The percentage of changes of anti-TB treatment was 61.54% in patients with mild hepatotoxicity and 82.93% in patients with severe hepatotoxicity, and the difference between the two groups was statistically significant (P = 0.02).

When analyzing the impact of ATLI on anti-TB treatment, three patients had to be excluded because they discontinued the treatment after the onset of ATLI and could not be followed up for TB treatment outcomes. Of the remaining 103 cases (94 unchanged and 9 discontinued anti-TB treatment cases), 53 (51.46%) cases had TB cured in time, 48 (46.60%) cased had therapy prolonged, and 2 (1.94%) died. In addition, recovery rate was 96.88% in 94 unchanged as compared to only 25.00% in 9 patients with discontinued anti-TB treatment patients (P<0.01).

Compared with non-ATLI patients, ATLI patients had 9.25-fold (95% CI, 5.69-15.05) increased risk of developing unsuccessful treatment outcomes and 2.11-fold (95% CI, 1.23–3.60) increased risk of prolonging intensive treatment phase (Table 5). PAR % for unsuccessful treatment outcomes and prolonged intensive treatment phase was 16.58% (95% CI, 10.16–25.30) and 2.57% (95% CI, 0.54–5.82) for ATLI and non-ATLI patients, respectively.

**Table 5:** Relative risk and attributable risk proportion for anti-TB treatment between ATLI and non-ATLI anti-TB treatment cases.^a^

		Result			
Category	Subcategory	ATLI	Non-ATLI	RR (95% CI)	AR %^d^ (95% CI)
Intensive treatment phase^b^	Prolonged	17 (16.19%)	323 (7.71%)	2.11 (1.23, 3.60)	52.51 (18.81, 72.22)
	Un-prolonged	88 (83.81%)	3864 (92.29%)		
Smear result at 2 months^c^	Negative converted	94 (91.26%)	3929 (94.54%)	1.56 (0.77, 3.17)	35.91 (−30.35, 68.48)
	Un-negative converted	9 (9.74%)	227 (5.46%)		
Clinical treatment outcome	Unsuccessful outcomes^e^	16 (15.09%)	67 (1.60%)	9.25 (5.69,15.05)	89.19 (82.42, 93.36)
	Successful outcomes^f^	90 (84.91%)	4131 (98.40%)		

a Abbreviation used in table: TB, tuberculosis; ATLI, Anti-tuberculosis Drug Induced Liver Injury; RR, relative risk; AR%: attributable risk proportion.

b Intensive treatment phase: the number of patients was 4292, because 12 patients missed data.

c Smear result at 2 months: the number of patients was 4259, because 45 patients missed smear examination.

d AR%: it is the percent of the incidence of an outcome in the exposed that is due to the exposure.

e Unsuccessful outcomes: A sum of treatment failure, died, default and transfer out.

f Successful outcomes: A sum of cure and treatment completed.

## Discussion

To our knowledge, this is the first multi-center population-based prospective study of ATLI to provide data on the incidence, clinical features and its impact on anti-TB treatment. The study population represented one of the largest and most diverse cohorts of TB patients receiving WHO recommended anti-TB treatment.

### Incidence of ATLI

Based on ATLI diagnosis criteria, the present study found a 2.55% cumulative incidence of ATLI in Chinese population receiving standard DOTS anti-TB treatment. This was lower than the rates reported in previously published studies that applied similar hepatotoxicity definitions, namely, 3.0% in Canada (Asia population accounted for 42%) [6], 5.0% in Hong Kong [15], 5.3% in Singapore [16] and 16.1% in Taiwan [17]. The lower ATLI incidence may be attributed to the study setting. Our study was population-based while others were hospital-based [6], [15], [17]. In hospital-based studies, the participants were likely to have more complex and severe diseases and were monitored more frequently, thus they had greater chances to reveal hepatotoxicity. Moreover, the ethnicity and genetics might play an important role. The Canada study [6] included non-Asia populations, having different genetic background. Several genetic polymorphisms in drug metabolizing enzymes were associated with ATLI, such as slow acetylator satus (N-acetyl-transferase 2), a glutathione S-transferase M1 homozygote mull genotype and cytochrome P4502E1c1/c1 genotype [18]. Thirdly, the prevalence of the virus infection also influences the incidence of ATLI, such as HIV and HCV. Literature suggests that the relative risk of developing drug-induced hepatitis was fivefold for hepatitis C patients, fourfold for HIV positive patients, and 14.4-fold for patients co-infected with hepatitis C and HIV. Unfortunately, our studies did not test HIV and HCV for every participant [19].

It should be noted that the cumulative incidence of ATLI was 1.84% in this study when only using the hepatotoxicity definition of ATS [14]. According to ATS, the hepatotoxicity definition does not include AST elevation. Moreover, asymptomatic individuals with ALT>3–5 times ULN are not considered to have hepatotoxicity. Actually, there are many definitions for drug-induced hepatotoxicity in various journals [7], [14]–[17]. In order to enable our study to be comparable with previous studies, we used the most frequently cited definition in those studies. In particular, the hepatotoxicity is defined as a treatment-emergent increase in transaminase greater than three or five times the upper limit of the normal, with or without symptoms of hepatitis, respectively.

### Clinical features of ATLI

71.59% of patients in this study developed ATLI during the first 2-month period after the treatment initiation, the median time elapsed from the initiation of anti-TB treatment to ALT elevation was 52.50 days. The median onset time of ATLI was longer as compared to what was reported in hospitalized TB patients. For example, a median onset day of 14 days and 15 days was reported in an Iran study [20] and a Turkey study [21]. Their results might be more receivable because hospitalized patients were visited and monitored more closely and precisely. The median onset time reported in our study was also longer than the 38 days reported in a community-based cohort study in Singapore [16]. The true ATLI onset time might be earlier than our estimation, because the current median days of detection could be impacted by the timing of ALT, AST and TBil tests during 1 to 2 months. In fact, 58.5% of the patients had the 2nd test 1 month after treatment initiation and 41.5% had it 1 month within treatment initiation.

Besides, our study indicated many ATLI patients experienced nausea, anorexia and vomiting. Although these symptoms are not specific to ATLI diagnosis [22], they might help clinicians be more cautious about ATLI. This could be helpful to clinicians working in developing countries, where ATLI diagnosis often relies on observation of clinical symptoms, as laboratory test may not be available [7]. On the other hand, other studies suggested that asymptomatic transaminase elevations occur in 20% of patients treated with standard anti-TB regimens [23]–[25], while in our study, 33.02% of ATLI patients, including 8 severe hepatotoxicity, did not present any clinical symptoms. Liver injury could be fatal when it is not recognized early or therapy is not interrupted in time [7]. Therefore, if available, routine monthly liver function monitoring is highly suggested for patients receiving anti-TB treatment in order to identify asymptomatic ATLI so as to apply appropriate intervention in time. However, monthly liver function monitoring is costly and could be difficult to perform among all TB patients in China, a more practical approach is to identify individuals who present some risk factors that are known to cause ATLI and monitor them closely. As revealed by literature [7], [8], [14], risk factors for ATLI include advanced age (above 60 years), female sex, malnutrition, HIV-infection, pre-existent liver disease, alcohol abuse, and concomitant use of other hepatotoxic drugs.

### Impact of ATLI on anti-TB treatment

Adherence to DOTS is critical in managing patients with active TB, because any change of anti-TB treatment will result in a suboptimal treatment response, and further reduce treatment effectiveness [7]. In our study, 69.81% of ATLI patients had to change their anti-TB treatment. Moreover, the magnitude of impact on the anti-TB treatment seemed to be related to the severity of the event. For instance, the percentage of changes of anti-TB treatment increased from 61.54% in mild hepatotoxicity patients to 82.93% in severe hepatotoxicity patients. 16.58% of unsuccessful treatment outcomes in our study might be attributed to ATLI. That is to say, if we could prevent the occurrence of ATLI, the percentage of patients with unsuccessful treatment outcomes would be decreased by 16.58%. Given that 1,000,000 new TB cases arise in China every year, over 25,000 patients might develop ATLI according to the ATLI incidence estimate in this study. If we could eliminate impact of ATLI, we would have prevented 4,228 TB patients from unsuccessful treatment outcomes. According to the WHO report, one patient remaining in mycobacterium transmittable status could possibly infect 10 to 15 more people in 12 months [2]. Therefore, preventing and minimizing the negative impact of ATLI to enhance the rate of successful TB treatment plays an important role in the overall efforts of the TB epidemic control in China.

Timely identification and appropriate intervention of ATLI are important. The guidelines for management of ATLI [26]–[28] advise TB patients to be treated under supervision and to examine liver function at signs or symptoms of hepatotoxicity. When ATLI diagnosis is confirmed, TB treatment should be interrupted in a timly manner and reintroduced until after the ATLI is resolved. There is no explicit suggestion to TB patients in China. Therefore more studies are needed to discuss the pattern of abnormal liver function, including onset time and frequency, to provide more evidence to develop practical management guidelines of ATLI.

### Strength and limitation

The major strength of this study included the large sample size and the well-established ADACS follow-up processes, which enabled us to estimate the incidence rate of ATLI and more importantly generalize the results to similar populations under certain conditions. In addition, diagnosis of ATLI is difficult because it requires the ruling out of other possibilities such as viral hepatitis or other possible causes of hepatotoxicity. The causality assessment was rarely conducted in previous studies in China, which might have lead to the misclassification of diagnosis. By contrast, in this study, the criteria for ATLI diagnosis and severity assessment were based on the main concepts of ATS and WHO. Each suspected ATLI case was strictly reviewed and assessed by experts from CDR to ensure the accuracy of ATLI diagnosis. Thirdly, this study reported and analyzed for the first time the impact of ATLI on anti-TB treatment based on a large-scale population-based cohort.

There were a few limitations in our study. Firstly, there were 1,817 non-responders who met the criteria for participation and 129 patients who dropped out during the monitoring period. The major reasons for non-responders were due to job relocation and subjects who could not continue to participate in the study. However, when comparing the difference in terms of age and sex among the 4,304 remainders, the 1,817 non-responders and 129 patients who dropped out of the study were no statistically significant. Secondly, although ad-hoc tests were given to patients who are suspected of having ATLI, the two routine tests might limit the ability of the study to detect ATLI, in addition to detecting it earlier. Thirdly, the study did not collect patients' information on prior history of hepatitis C infection and alcohol consumption, which are risk factors for hepatotoxicity.

### Conclusion

This is the first large-scale population-based cohort study to estimate the incidence and impact of ATLI in China. The study indicated that the cumulative incidence of ATLI is 2.55% in Chinese TB patients receiving DOTS treatment. ATLI could considerably impact the anti-TB treatment, potentially leading to unsuccessful treatment outcomes and the prolongation of intensive treatment phase. Furthermore, one-third of ATLI patients were found with no symptoms, suggesting that monitoring liver function and identifying known risk factors of ATLI in addition to the symptoms themselves are critical supplements in diagnosing patients with ATLI. Given the incidence of ATLI and the size of the TB population in China, the negative impact of ATLI on anti-TB treatment is substantial. Therefore, more research and efforts are warranted in order to enhance the diagnosis and the prevention of ATLI.
